# Solid-Phase Primer Elongation Using Biotinylated dNTPs for the Detection of a Single Nucleotide Polymorphism from a Fingerprick Blood Sample

**DOI:** 10.1021/acs.analchem.1c03419

**Published:** 2021-10-27

**Authors:** Miriam Jauset-Rubio, Mayreli Ortiz, Ciara K. O’Sullivan

**Affiliations:** † INTERFIBIO Research Group, Departament d’Enginyeria Química, Universitat Rovira i Virgili, Avinguda Països Catalans 26, 43007 Tarragona, Spain; ‡ InstitucióCatalana de Recerca i Estudis Avancats (ICREA), Passeig Lluís Companys 23, 08010 Barcelona, Spain

## Abstract

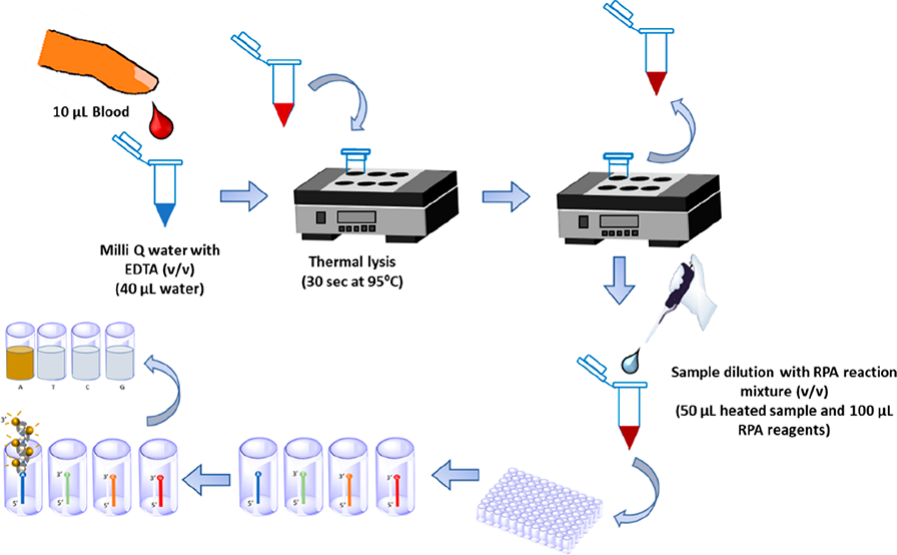

Isothermal recombinase polymerase amplification-based solid-phase primer extension is used for the optical detection of a hypertrophic cardiomyopathy associated single nucleotide polymorphism (SNP) in a fingerprick blood sample. The assay exploits four thiolated primers which have the same sequences with the exception of the 3′-terminal base. Target DNA containing the SNP site hybridizes to all four of the immobilized probes, with primer extension only taking place from the primer containing the terminal base that is complementary to the SNP under interrogation. Biotinylated deoxynucleotide triphosphates are used in the primer extension, allowing postextension addition of streptavidin-poly-horseradish peroxidase to bind to the incorporated biotinylated dNTPs. The signal generated following substrate addition can then be measured optically. The percentage of biotinylated dNTPs and the duration of primer extension is optimized and the system applied to the identification of a SNP in a fingerprick blood sample. A methodology of thermal lysis using a 1 in 5 dilution of the fingerprick blood sample prior to application of 95 °C for 30 s is used to extract genomic DNA, which is directly used as a template for solid-phase primer extension on microtiter plates, followed by optical detection. The SNP in the fingerprick sample was identified and its identity corroborated using ion torrent next generation sequencing. Ongoing work is focused on extension to the multiplexed detection of SNPs in fingerprick and other biological samples.

In the emerging era of pharmacogenetics and personalized medicine, there is an increasing need for nucleic acid analytical tools that are inexpensive and simple to use and which can be used at the point-of-care with minimal sample processing requirements. Nucleic acids are typically amplified using the polymerase chain reaction (PCR), which requires thermal cycling, and while portable microsystems capable of carrying out PCR have been developed, a more attractive solution is the use of isothermal amplification, including nucleic acid sequence-based amplification (NASBA), strand displacement amplification (SDA), rolling circle amplification (RCA), loop-mediated isothermal amplification (LAMP), and helicase-dependent amplification (HDA), as well as recombinase polymerase amplification (RPA). RPA is notable due to its ease-of-use, high sensitivity, selectivity, compatibility with multiplexing, rapid amplification, the use of a low and constant temperature with no need for tight temperature control, as well as not requiring an initial denaturation step or the use of multiple primers. Furthermore, in contrast to many polymerases used in PCR, RPA is not inhibited by blood components, and extracted genomic DNA can be amplified in the presence of the blood matrix.^1^ Overall, RPA is highly attractive for use at the point-of-care or point-of-need.^2−5^

RPA starts when a recombinase protein binds to primers in the presence of ATP (adenosine triphosphate) and a high molecular polyethylene glycol “crowding agent,” forming a recombinase–primer complex. The complex then seeks a homologous sequence in the DNA being interrogated, forming a D-loop at the cognate site. The displaced DNA strand is stabilized by single-stranded binding proteins, and the recombinase then disassembles and a strand displacing DNA polymerase binds to the 3′ end of the primer and elongation is initiated.^1,6,7^ Solid-phase RPA has also been reported, where one of the two primers is immobilized on a substrate and the other is maintained in the solution phase, resulting in surface-tethered specifically amplified DNA targets.^8−10^ The amplified DNA can then be detected using a variety of different transduction methodologies, including electrochemical and chemiluminescence detection.^11,12^ Solid-phase RPA is particularly attractive due to its compatibility with multiplexed amplification and detection.^13^ Solid-phase RPA offers significant advantages over solid-phase PCR, where specifically designed thermostable surface chemistries are required, and often the immobilized primers desorb from the surface as they cannot withstand the high temperatures applied, introducing problems of irreproducibility.^14^ Solid-phase amplification can potentially also overcome problems with primer-dimers, which can be encountered with solution-phase RPA. A variety of surfaces have been used for solid-phase RPA, including polystyrene microtiter plate, silicon, gold electrodes, and indium tin oxide electrodes, among others.^1^

Different approaches have been developed for the detection of solid phase amplification products, including the use of biotin labeled solution phase primer, followed by the addition of enzyme labeled streptavidin,^15^ or alternatively the use of enzyme labeled primer^15,16^ or redox decorated nanoparticle labeled primer.^17^

An alternative approach is the use of biotinylated deoxynucleotide triphosphates (biotin-dNTPs), which should lead to an enhancement in signal due to the increased number of biotin labels incorporated. In 1981, Langer reported the chemical synthesis of biotinylated dUTPs and showed them to be effective substrates for a variety of polymerases,^18^ and soon after Brigati et al. prepared biotinylated dNTPs with different length linkers,^19^ with the same group showing that DNA probes labeled with biotin-11-dUTP of biotin-16-dUTP could be detected via avidin-linked alkaline phosphatase.^20^ The synthesis of biotinylated dATP and dCTP was subsequently reported,^21,22^ as well as dGTP.^23^ Since then, a range of biotinylated dNTPs with various linkers have been synthesized, characterized, and used in a plethora of applications, including sequencing,^24^ biosensors,^25−28^ dipsticks.^29,30^

While the majority of reports of isothermally amplified biotinylated products use biotin-labeled primers rather than dNTPs, there are some examples, such as that reported by Jung et al. in 2015, where biotin-dUTP was used in multiplexed LAMP and the products detected using an immunochromatographic strip.^31^ The use of RCA and biotinylated nucleosides has also been reported, such as the technique described by Xu et al., where they exploited a positively charged nylon membrane for nucleic acid immobilization and near-infrared fluorescence for the detection of the biotinylated amplicon.^32^ Zhan et al. used an alternative approach using RCA, for the attomolar detection of exosomal miRNA based on the use of a terahertz metamaterial comprised of an array of gold (Au) discs.^33^

There are a few reports of the combination of biotinylated-dNTPs and RPA, with the first being by Trau’s team in 2016, who developed a system for the detection of TMPRSS2-ERG, the most frequent fusion gene, associated with prostate cancer. In their assay, mRNA was reverse transcribed and amplified with RPA using biotinylated dUTPs, and the product could finally be visualized by the naked eye. They also developed an electrochemical detection platform and used the assay to identify the source of the fusion gene in urine,^34^ as well as used a similar approach using gold nanoparticles and SERS detection.^35^ The same team went on to describe a similar optical/electrochemical system using screen-printed electrodes for the detection of *Mycobacterium tuberculosis*.^36^ Li et al. detailed an isothermal paper biosensor, using a single universal primer and biotin-dCTPs for the multiplex detection of genetically modified maize.^37^ Kortli et al. reported on the use of biotinylated dNTPs with RPA, for the detection of *Yersinia pestis* using a lateral flow assay, achieving a detection limit of 7 fM and 0.63 fg achieved for synthetic and genomic DNA, respectively.^38^

In the work reported here, the aim was to combine solid-phase primer extension mediated via RPA with biotinylated dNTPs for the colorimetric identification of a single nucleotide polymorphism (SNP). A SNP related with hypertrophic cardiomyopathy, with the targeted SNP located in the 14q12 locus of the human β-myosin heavy chain (MYH7), was used as a model system.^39,40^

Four 5′ thiolated reverse primers, identical with the exception of the 3′ terminal base, were immobilized on the wells of a maleimide activated microtiter plate for colorimetric detection. Double stranded synthetic/genomic DNA was added to the microtiter plate and allowed to hybridize to the four primers, with full complementarity between probe and target DNA only obtained with the primer, with the terminal base complementary to the SNP present in the DNA being interrogated. Primer elongation via solid-phase RPA using biotinylated dNTPs was only observed with the fully complementary primer. Streptavidin-poly-horseradish peroxidase (SA-poly-HRP) was added following isothermal amplification and the colorimetric signal measured following addition of the enzyme substrate. To demonstrate the possibility of implementing the assay to the point-of-care (POC), the SNP was detected directly from a fingerprick blood sample. In order to achieve this goal, we explored methods of RPA-compatible cell lysis, which could be implemented in a POC setting. Finally, we validated the identification of the SNP present in the human fingerprick blood sample using next generation sequencing.

## Materials and Methods

### Materials

Phosphate-buffered saline (PBS; 10 mM phosphate, 137 mM NaCl, 2.7 mM KCl, pH 7.4), PBS-Tween (10 mM phosphate, 137 mM NaCl, 2.7 mM KCl, 0.05% v/v Tween 20, pH 7.4), Triton X-100, skimmed milk powder, 3,3′,5,5′-tetramethylbenzidine (TMB), and all other reagents were purchased from Sigma (Barcelona, Spain). Maleimide activated plates, eight-well strips, *Dream taq* DNA polymerase, proteinase K, and Gene Ruler DNA ladder were from Fischer Scientific (Madrid, Spain). Certified Low Range Ultra Agarose was purchased from Bio-Rad (Barcelona, Spain). Streptavidin Poly-HRP80 was supplied from SDT-reagents (Baesweiler, Germany). Biotin-16-dCTP and Biotin-16-dUTP were purchased from Jena Bioscience (Jena, Germany). All DNA oligonucleotides were purchased from Biomers (Germany). All solutions were prepared in high-purity water obtained from the Milli-Q RG system (Barcelona, Spain).

### Primer Design

Primers and synthetic sequences for the detection of SNP rs743567 found in MYH7 gene (*Homo sapiens* beta-myosin heavy chain gene) were designed to be specific using Primer Blast and Multiple Primer Analyzer (ThermoFischer Scientific) tools. The PCR synthesis of double stranded DNA to be used as a model template to mimic the genome is described in the SI. All primer and probe sequences can be found in Table S-1.

### Genomic DNA Extraction

#### Chemical Lysis

The chemical lysis buffer (125 mM NaOH, 1% Triton-X-100, 1 mg/mL proteinase K) was prepared as previously described by Eid et al.,^41^ with some minor modifications. The lysis procedure was carried out as follows: 10 μL of finger-prick blood sample was mixed with 10 μL of lysis buffer and incubated for 4 min at 25 °C. In order to evaluate the extraction yield and the coagulation effect, the assay was also performed in the presence and absence of EDTA (final concentration of 5 mM) as well as diluting the blood sample 1 in 2, prior to mixing with the lysis buffer. PCR and RPA were carried out with the extracted DNA.

PCR was carried out as follows: 5 μL of the treated blood sample was added to 45 μL of master mix reagents containing 1 × Dream Taq buffer, 200 nM of primers, 0.2 μM dNTPs, and 1 U Dream Taq polymerase. As a positive control, 100 pM of synthetic DNA was used. The program used was 95 °C for 2 min, followed by 25 rounds of PCR with 30 s of denaturation at 95 °C, 30 s of annealing at 60 °C, and 30 s of elongation at 72 °C, with a final extension step at 72 °C for 5 min.

RPA was performed according to the manufacturer’s instructions (TwistAmp Liquid Basic kit, TwistDx, Cambridge, UK). Briefly, 5 μL of the treated blood sample was added to 45 μL of RPA reagents (1 × rehydration buffer, 1 × basic E-mix, 1 × core reaction mix, 0.5 μM of primers, 0.2 mM dNTPs, 10 mM Mg(OAc)_2_) and incubated for 15 min at 37 °C. For positive control, 100 pM of synthetic DNA was used. Before running the samples in a 2.6% agarose gel electrophoresis and stopping the RPA reaction, the samples were heated at 80 °C for 10 min.

Five microliters of PCR/RPA amplified products was mixed with 4 μL of 6 × loading buffer and run in 2.6% (w/v) AGE prepared in 1 × TBE buffer (Tris-Borate-EDTA, pH 8) at 110 mV for 20 min. The gel was prestained with GelRed nucleic acid stain (VWR, Spain) and imaged with a UV lamp (λ = 254 nm).

#### Thermal Lysis

Ten microliters of a fingerprick blood sample was diluted using a defined dilution factor in sterilized Milli-Q water containing 5 mM EDTA and heated to 95 °C for a defined amount of time. To optimize parameters and avoid any coagulation effect, a dilution factor of the blood sample (undiluted, dil. 1 in 2, dil. 1 in 5, and dil. 1 in 10) and the heating time (10 s, 30 s, 60 and 120 s) were tested.

### SNP Detection on Microtiter Plate Using Solid-Phase Primer Extension

A solid-phase primer extension assay was performed from primers immobilized on the surface of the wells of a maleimide activated microtiter plate. One hundred microliters of thiolated reverse primers (200 nM in PBS) were added to the wells of the microtiter plates and left to incubate overnight at 4 °C. The wells were washed with 200 μL of PBS-Tween and blocked with 200 μL of 5% w/v skimmed milk in PBS-Tween for 1 h at 22 °C. After washing the wells again with PBS-tween, 50 μL of RPA reagents (1x Rehydration buffer, 1x Basic E-mix, 1x Core Reaction mix, 0.5 μM of Fw primer, 0.2 mM dNTPs (containing dATP, dGTP, dTTP with 20% of biotinylated-dCTP/dCTP), 10 mM Mg(OAc)_2_ and 1 nM synthetic dsDNA template for optimization, or different target concentrations for the calibration curve from 1 fM to 25 nM) was added to each well and incubated at 37 °C for 15 min. Two-hundred microliters of 100 mM NaOH was added to each well for 2 min in order to remove/denature any remaining proteins from the RPA reaction. SA-poly-HRP80 (50 μL of dilution 1 in 20 000 of 1 mg/mL) in PBS-tween was added, and the plate was allowed to incubate for a further 30 min. Finally, the wells were washed with PBS-Tween, and 50 μL of TMB substrate was added for 5 min, followed by the addition of an equal volume of 1 M H_2_SO_4._ The absorbance was read at 450 nm.

For the detection of the SNP in the fingerprick blood sample, the same procedure of thermal lysis was followed as explained above, using dilution 1 in 5 in Milli Q water containing 5 mM EDTA and heated for 30 s at 95 °C. Five microliters of extracted DNA was added to the 45 μL of RPA reagents per well.

### Next Generation Sequencing

The genomic DNA template extracted after thermal lysis was amplified using the same conditions used for PCR. Modified primers, containing barcode sequences, were used for the amplification (Table S-1). The resulting amplicon was column-purified using a DNA Clean and Concentrator kit (Ecogen, Spain) and, subsequently, sequenced using Ion Torrent Next-Generation Sequencing (NGS) in the COS facility (Centre for Omic Sciences, Eurecat Technology Centre, Reus, Spain).

FastaQ raw data were imported into the Galaxy web server for analysis (https://usegalaxy.org). The format of the data was then converted to FASTA using the “FASTAQ to FASTA converter” tool. The length of the sequences was filtered to the expected length of the amplified product (80 bp) using the option of “Filter sequences by length” in order to remove amplification and sequencing artifacts from the data sets. Unique sequences were then identified using the “Collapse” tool. The most representative sequence was chosen as the correct sequence for SNP identification.

## Results and Discussion

### SNP Detection on Microtiter Plate Using Solid-Phase Primer Extension and Synthetic dsDNA

**Figure 1:**
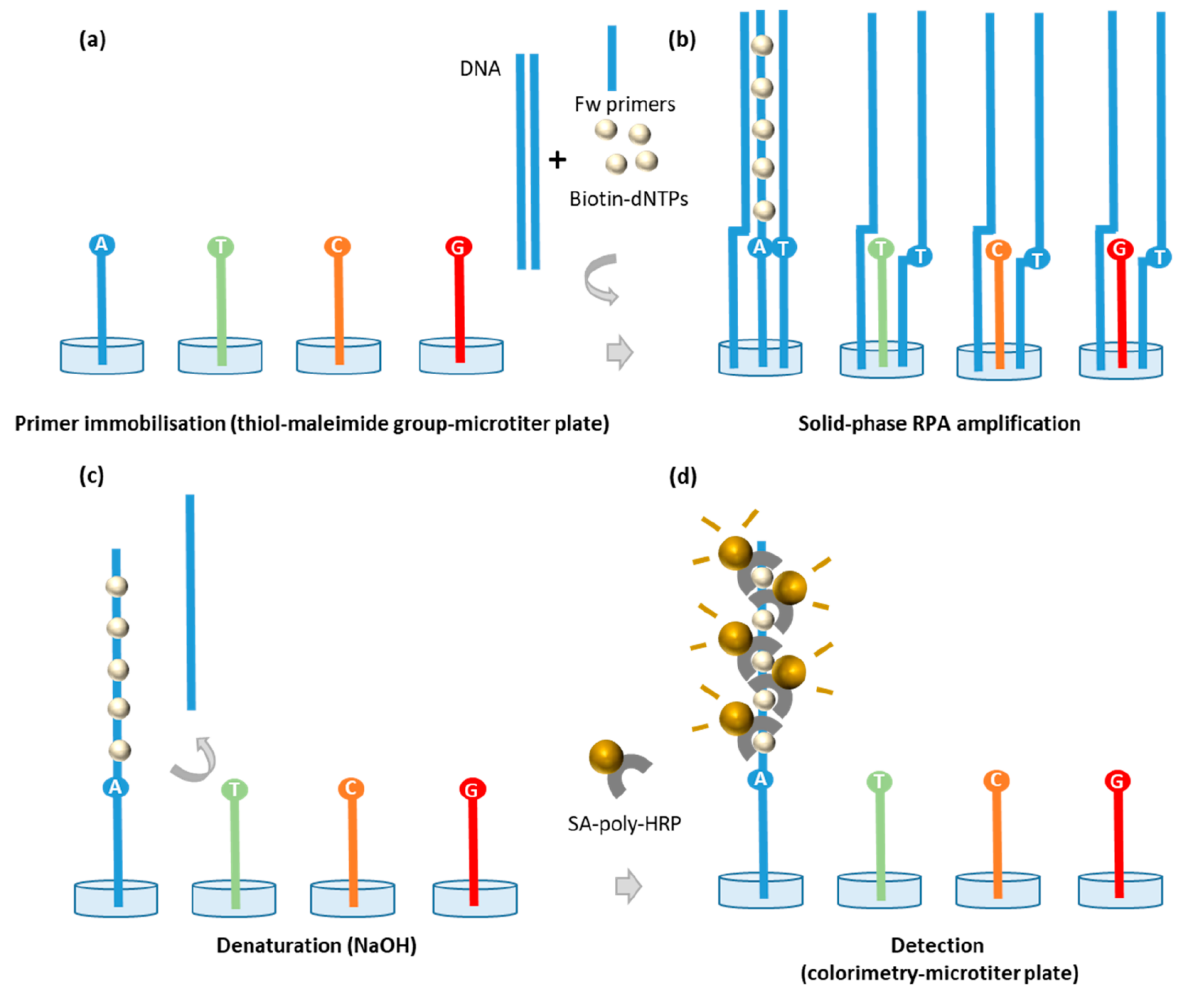
Schematic representation of the steps of the solid-phase amplification assay on a microtiter plate: (a) thiol-primer immobilization on solid support, the four different primers were designed to be identical and complementary to the target up to the base adjacent to the polymorphic site located at the 3′ end were immobilized on individual wells of a microtiter plate; (b) solid phase RPA amplification, the target genomic DNA hybridizes to the primers and starts the solid-phase amplification and primer elongation only for the fully complementary primer; (c) denaturation of the surface tethered amplified dsDNA with NaOH, denaturing the elongated duplex, while also removing any components of the RPA mix that have adsorbed to the surface of the wells of the microtiter plate; and finally, (d) the addition of SA-poly-HRP, SA-poly-HRP binds to the biotinylated dNTPs incorporated in the amplified DNA for colorimetric detection following addition of the TMB substrate.

Optimization of the percentage of the biotinylated/natural dNTPs and duration of the solid-phase primer extension to facilitate the most robust identification of the SNP under interrogation was primarily carried out using a synthetic double stranded DNA using a microtiter plate. Four thiolated reverse primers were immobilized on the surface of the wells of a maleimide microtiter plate, where the primers were designed to be identical and complementary to the target up to the base adjacent to the polymorphic site located at the 3′ end, where each primer had a different terminal base. Following hybridization with the target DNA primer extension should only occur at the primer where there is full complementarity, thus facilitating identification of the SNP under interrogation. Following primer extension, SA-poly-HRP was added, followed by the enzyme substrate, and the color produced was measured (Figure 1).

**Figure 2:**
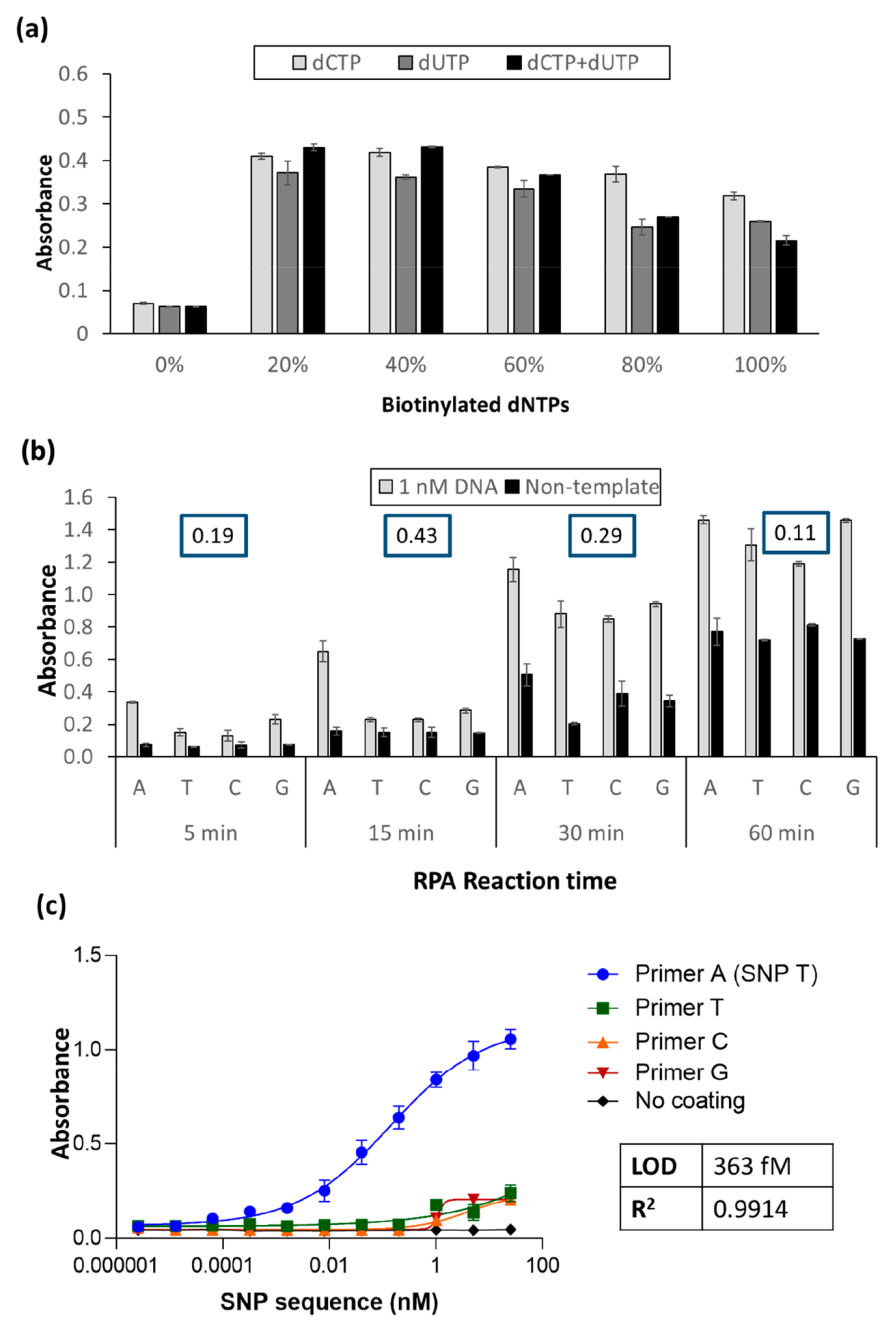
Optimization of (a) the percentage of biotin-dCTPs and biotin-dUTPs in the reaction mixture and (b) the reaction time of recombinase polymerase-mediated solid phase primer extension. The difference between the signal from the specific primer and the media of the nonspecific primer (absorbance (PA)_specific-primer_ – (Absorbance (PT + PC + PG)_nonspecific-primer_/3]) is reported in the insets for each assay time studied. (c) Calibration curve of solid-phase amplification using the optimized conditions (20% dCTPs and 15 min primer elongation).

Double stranded synthetic DNA was produced using PCR to mimic the genomic DNA and was used for these optimization studies. As can be seen in Figure 2a, using a 10 min amplification time, when comparing ratios of biotinylated dCTP and dUTP alone, with a combination of the two, similar results were obtained using 20% or 40% of the individual modified dNTPs, or a mixture of the dCTP and dUTP, with increasing percentages resulting in decreased absorbances, which can be attributed to an inhibition of the RPA. In addition, the combination of the dCTP and dUTP did not significantly improve the signal and thus 20% of dCTPs were used for the optimization of the amplification time. Increasing amplification time was observed to minimize the difference in signal between the correct SNP and also to increase the nonspecific signal obtained with the nontemplate control. Increasing amplification times could allow the nonspecific primer to eventually be elongated, and these longer amplification times also result in a higher degree of nonspecific adsorption of the RPA proteins on the surface of the maleimide plate, which in turn results in nonspecific adsorption of the SA-poly-HRP level. This latter effect was easily addressed via a rapid basic pH wash (Figure 1c), where the nonspecifically adsorbed proteins are denatured/removed and the signal from the nontemplate controls was drastically reduced (Figure S-1b) as compared with Figure 2b.

As observed in the inset of Figure 2b, the highest value of the specific signal obtained with the primer bearing the terminal base complementary to the base minus the average of the signals from the other three primers was obtained using a 15 min primer elongation, where an unequivocal SNP discrimination was obtained. Fifteen minutes of amplification was thus deemed optimal. To corroborate the results obtained using solid phase amplification, an evaluation of liquid-phase primer elongation time was carried out, and the corresponding electrophoresis gels are shown in Figure S-1a. As observed in the agarose gels, the maximum differentiation between positive and negative signals was again obtained using a 15 min primer elongation. A longer reaction time results in a lower differentiation between signals and does not allow a clear identification of the SNP.

Using the optimal conditions, a calibration curve was carried out to determine the limit of detection (LOD) of the assay and evaluate its further application to real samples. As observed in Figure 2c, the signal generated from the fully complementary primer increased with increasing target concentration, while negligible signals were observed with the other three primers and the no coating controls. A limit of detection of 363 fM was achieved.

### Genomic DNA Extraction from Fingerprick Blood Sample

We initially evaluated the use of chemical lysis using solution phase RPA and PCR; the amplification yield achieved using RPA was markedly lower than that of PCR (Figure S-2). We thus explored the possibility of using thermal lysis to see if this method could result in similar amplification yields with RPA as chemical lysis with PCR. Thermal lysis is the earliest and simplest reagentless method of cell lysis, where the proteins of the cell membrane are denatured at temperatures of 90–100 °C, thus releasing the intracellular contents including genetic material. Despite its simplicity, and presumably due to problems of coagulation and protein precipitation, thermal lysis has not been extensively used with blood samples, although there are some examples such as the wireless induction heating mediated thermal lysis reported by Baek et al.^42^ and the printed circuit board device by Marshall et al.^43^

Increasing times of heating at 95 °C (10, 30, 60, and 120 s) and different dilution factors of blood in 5 mM EDTA (blood/EDTA v/v = 1:0 (undiluted, blood directly used); 1:2; 1:5 and 1:10) were evaluated in terms of coagulation (visual analysis, Figure S-3a) and further detection of the extracted DNA from a fingerprick sample (amplification yield by the intensity of the band in the gel electrophoresis after RPA reaction, Figure S-3b). Similar amplification yields were obtained with all the thermal lysis durations. However, longer thermal lysis times and/or lower dilution factors (e.g., undiluted or 1:2) resulted in blood coagulation. Optimum results in terms of amplification yield and absence of coagulation were achieved using solution phase RPA with the sample diluted 1:5 (v/v) in 5 mM EDTA and heated for 10–30 s (Figure S-3).

### SNP Detection on Microtiter Plate Using Solid-Phase Primer Extension from a Fingerprick Blood Sample

**Figure 3:**
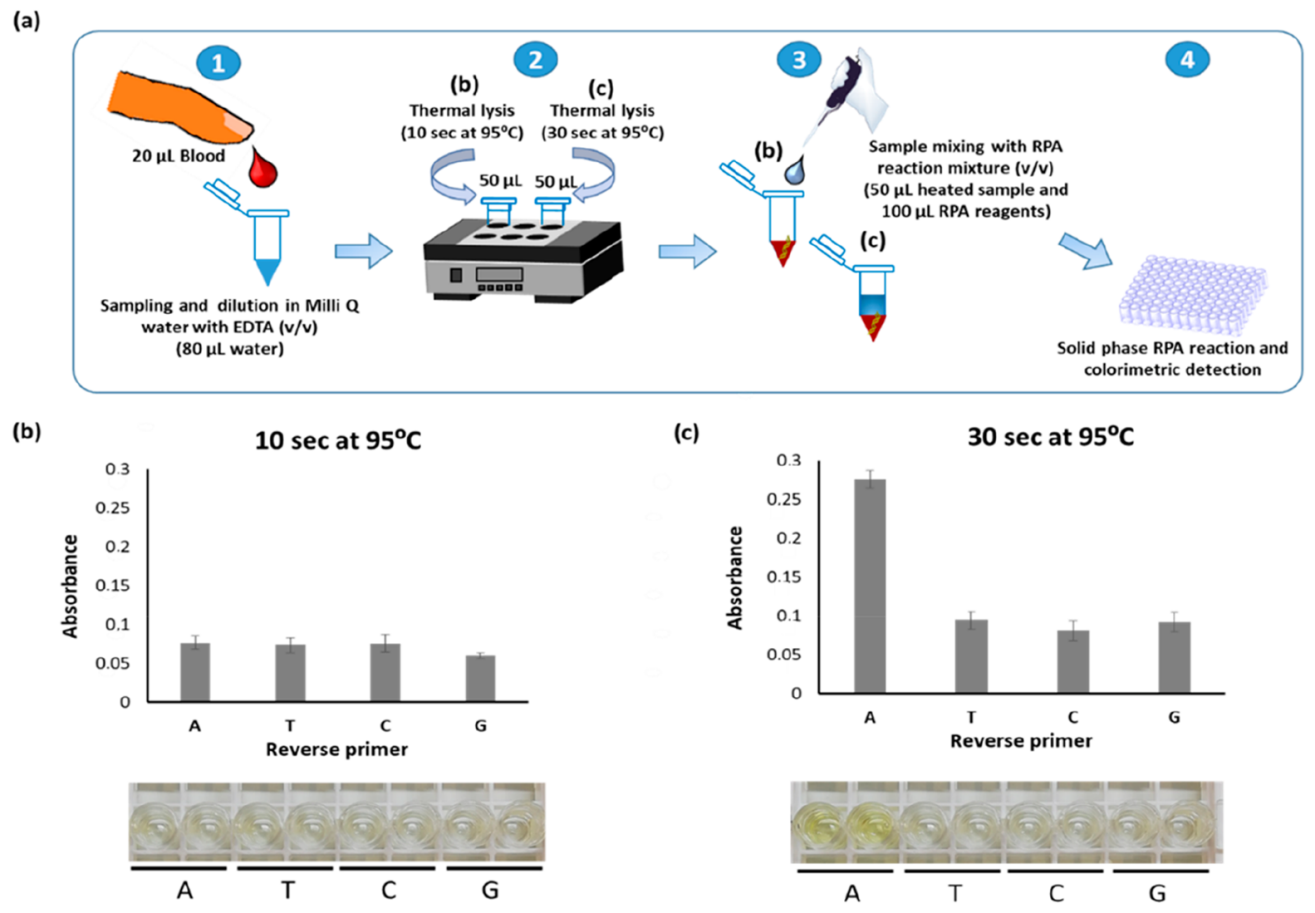
(a) Schematic representation of the whole assay, where two heating times (b, 10; c, 30 s) were compared for cell lysis, based on the SNP detection via solid-phase RPA reaction and colorimetric detection. (b and c) Absorbance recorded after RPA reaction using different heating times, (b) 10 s and (c) 30 s, at 95 °C and the corresponding real pictures of microtiter plates, to demonstrate the visual detection of the SNP.

The optimum parameters of thermal lysis (10–30 s with dilution 1 in 5 v/v in Milli-Q water containing 5 mM EDTA) previously tested in solution phase were applied in recombinase polymerase-mediated solid phase primer extension. Fingerprick blood was diluted accordingly and the solution divided into two vials, and each vial was heated to 95 °C for either 10 or 30 s. The extracted DNA was amplified using RPA with 20% biotin-dCTPs, a 15 min amplification time, and a 30 s thermal lysis time, observed to be better. As shown in Figure 3c, heating for 10 s was not adequate for efficient cell lysis and DNA extraction, while a clear difference was observed for primer A, which contains the terminal base complementary to the SNP (in this case T), when the thermal lysis was applied for 30 s.

In order to confirm the identity of the SNP in the fingerprick blood sample, ion-torrent next generation sequencing was performed. The genomic DNA was extracted by thermal lysis and amplified using PCR with modified primers containing specific barcodes for the sequencing (Table S-1). The data were analyzed by filtering it to the expected size (80 bp) and collapsed to identify the most representative sequences which contain the SNP. The sequencing results corroborated that SNP T is the SNP found in the blood sample (Tables S-2 and S-3).

In order to explore the effects of the blood matrix on the assay, the detection of a SNP in a nonhuman DNA sequence was used. A SNP associated with rifampicin resistance in *Mycobacterium tuberculosis* was used to carry out this study to probe the matrix effect. Four individual primers, again equivalent with the exception of the terminal 3′ base, were immobilized on individual electrodes. A synthetic DNA sequence spiked into either a PBS or fingerprick blood sample, and exposed to 30 s at at 95 °C, was mixed with RPA reagents, and the assay methodology was followed in the same manner as that carried out for the detection of the cardiomyopathy associated SNP. As can be seen in Figure S-4, no difference in signal was observed when carrying out the assay in a buffer or thermally lysed fingerprick blood, highlighting the lack of any matrix effect.

Finally, although the LOD of the present approach is higher than those reported for other methods (Table S-4),^44−49^ it demonstrated to be adequate for the unequivocal identification of a SNP from a fingerprick sample. Moreover, our method, which can be performed in less than 50 min (DNA extraction, 30 s; amplification, 15 min; SA-Poly HRP, 20 min; detection, 5 min) and under 37 °C isothermal conditions, achieves a considerable reduction in assay time, complexity, and costs when compared with other SNP detection methods as summarized in Tables S-4, S-5, and S-6.

## Conclusions

Isothermal recombinase polymerase amplification based solid-phase primer extension was used for the detection of a hypertrophic cardiomyopathy associated SNP in a fingerprick blood sample. The solid-phase primer extension was based on the use of four thiolated primers designed to be identical, with the exception of the 3′-terminal base. Double stranded DNA containing the SNP site to be interrogated was then added to hybridize to the immobilized probes, and the recombinase polymerase amplification initiated using a mixture of native dNTPs with dUTPs, dCTPs, or a combination of dUTPs and dCTPs. Primer extension should only take place from the primer containing the terminal base that is complementary to the SNP under interrogation; thus following the addition of SA-poly-HRP to bind to the incorporated biotinylated dNTPs and subsequent substrate addition, the signal should only be measured at one of the primers. For initial optimization of the assay, the primers were immobilized on the surface of maleimide microtiter plates. A range of percentages of biotinylated dCTP, dUTP, and a mixture of both were evaluated and the optimum observed to be either 20% biotinylated dCTP alone or a 20% mixture of biotinylated dCTP and biotinylated dUTP deemed optimal. The amplification time for the optimal differential signal was then evaluated and determined to be 15 min, with increasing amplification times resulting in a lower differential signal and increasing background. The assay was applied to the detection of cardiomyopathy SNP in a fingerprick blood sample. A method for RPA-compatible thermal lysis was developed, where simple heating of a fingerprick blood sample for 30 s at 95 °C resulted in an amplification yield of the thermally extracted DNA similar to that obtained using chemical lysis and PCR. Ongoing work is focused on extension to the multiplexed detection of SNPs in fingerprick and other biological samples.

## Supporting Information Available

Supplementary tables (sequences used on this work, next generation sequencing results; comparison of our approach with other SNP detection methods reported in the literature; detailed cost analysis of the present approach and prices of commercial kits for DNA extraction from blood), additional experimental details (double stranded DNA generation from synthetic ssDNA to mimic genomic DNA; assay time of the RPA reactions performed in liquid and solid phases; study of the blood matrix effect on the recombinase polymerase-mediated solid phase primer extension), and additional figures (assay time of the RPA reaction performed in liquid and solid phases; samples pretreated using chemical lysis buffer to extract the human genomic DNA and further amplified, a photograph showing the coagulation effect and the electrophoresis gels after PCR and RPA; optimization of heating time vs dilution factor in samples pretreated using thermal lysis to extract the human genomic DNA and further amplified, photographs showing the coagulation effect and the electrophoresis gels after RPA), and a study of the blood matrix effect on the RPA reaction (PDF)
