# Supplementary material for: Solid-Phase Primer Elongation Using Biotinylated dNTPs for the Detection of a Single Nucleotide Polymorphism from a Fingerprick Blood Sample
Source: Anal Chem. 2021 Oct 27;93(44):14578–85. doi: 10.1021/acs.analchem.1c03419 (PMC8581964; doi:10.1021/acs.analchem.1c03419)
Supplement: Supplementary file 1 — ac1c03419_si_001.pdf [file ac1c03419_si_001.pdf]

Supporting Information

Solid-phase primer elongation using biotinylated dNTPs for the detection of a single nucleotide polymorphism from a fingerprick blood sample

Miriam Jauset-Rubio[aŦ], Mayreli Ortiz[aŦ] and Ciara K. O'Sullivan[a,b*]

[a]INTERFIBIO Research Group, Departament d'Enginyeria Química, Universitat Rovira i Virgili, Avinguda Països Catalans 26, 43007 Tarragona, Spain

[b]Institució Catalana de Recerca i Estudis Avancats (ICREA), Passeig Lluís Companys 23, 08010 Barcelona, Spain

*Corresponding author: ciara.osullivan@urv.cat

Ŧ Equal contribution

Table of content

Table S-1. Sequences used in this work. 2

Table S-2. Next generation sequencing (NGS) results: Basic Statistics. 3

Table S-3. Next Generation Sequencing (NGS) results: Top 10 sequences. 4

Table S-4. Comparison of our approach with other SNP detection methods reported in literature. 5

Table S-5. Detailed cost analysis of the present approach 7

Table S-6. Prices of commercial kits for DNA extraction from blood 7

Supplementary Text 8

1. Double stranded DNA (dsDNA) generation from synthetic ssDNA to mimic genomic DNA 8
2. Assay time of the RPA reactions performed in liquid and solid phases 8
3. Study of the blood matrix effect on the recombinase polymerase-mediated solid phase primer extension. 9

Figure S-1: Assay time of the RPA reaction performed in (a) liquid and (b) solid phases. (a) The 2.6% agarose electrophoresis gels after the RPA reaction at different times show the increment of the intensity of the bands from non-specific primers with the reaction time. (b) The absorbance values recorded after recombinase polymerase-mediated solid phase primer extension also increase with the reaction time. The non-template controls were reduced respect to those reported in Figure 2b by introducing a treatment at basic pH. (The difference between the signal from the specific primer and the media of the non-specific primer (Absorbance $(PA)_{Specific\ primer}$ - (Absorbance $(PT+PC+PG)_{Nonspecific\ primer}$ /3]) is reported in the insets for each time. 10

Figure S-2: Samples pre-treated using chemical lysis buffer to extract the human genomic DNA and further amplified: (a) A photograph showing the coagulation effect; electrophoresis gels after (b) PCR; (c) RPA. 11

Figure S-3: Optimisation of heating time vs. dilution factor in samples pre-treated using thermal lysis to extract the human genomic DNA and further amplified: (a) Photographs showing the coagulation effect; (b) electrophoresis gels after RPA. 11

Figure S-4: Study of the blood matrix effect on the RPA reaction. RPA reaction was performed using a target sequence that is non present in human genome (from *Mycobacterium tuberculosis*) spiked in PBS an in a blood sample. Positive primer G (SNP C). 12

References: 12

Table S-1. Sequences used in this work.

**DNA Extraction**	**Sequences**
Synthetic ssDNA (80b)	5'-CAAAGAAATGGCCCTGATCCCCTGACTTCTT**T**AATGTGAGGGCATTTCTTGGTGCCCTGTGGGCTCTCTGTGAAAAGCCA-3'
Fw primer (21b)	5'-CAAAGAAATGGCCCTGATCCC-3'
Rev primer (20b)	5'-TGGCTTTTCACAGAGAGCCC-3'
**Solid-phase amplification**	**Sequences**
Synthetic ssDNA (130b)	5'-ACTGTTTACCAAGTCTGGAAAGTCTATACATTTTGGAAGAACTTACACTAAGAATTGGAACCACAAGTTAAGCAAAACAAAGAAATGGCCCTGATCCCCTGACTTCT**T**TAATGTGAGGGCATTTCTTGGT-3'
Fw primer (25b)	5'-ACTGTTTACCAAGTCTGGAAAGTCT-3'
Thiol Rev primer A (37b)	5'-Thiol-C6-TTTTTTTTTTTTTTTTACCAAGAAATGCCCTCACATT**A**-3'
Thiol Rev primer T (37b)	5'-Thiol-C6-TTTTTTTTTTTTTTTTACCAAGAAATGCCCTCACATT**T**-3'
Thiol Rev primer C (37b)	5'-Thiol-C6-TTTTTTTTTTTTTTTTACCAAGAAATGCCCTCACATT**C**-3'
Thiol Rev primer G (37b)	5'-Thiol-C6-TTTTTTTTTTTTTTTTACCAAGAAATGCCCTCACATT**G**-3'

NGS	Sequences
Synthetic ssDNA (80b)	5’-CAAAGAAATGGCCCTGATCCCCTGACTTCTTTAATGTGAGGGCATTTCTTGGTGCCCTGTGGGCTCTCTGTGAAAAGCCA-3’
Fw primer (65b)	5’-CCATCTCATCCCTGCGTGTCTCCGACTCAGTCAGTCCGAACGATCAAAGAAATGGCCCTGATCCC-3’
Rev primer (45b)	5’-CCTCTCTATGGGCAGTCGGTGATCCTGGCTTTTCACAGAGAGCCC-3’

*Primer regions are underlined in black. Barcodes for NGS are underlined in red.

Table S-2. Next generation sequencing (NGS) results: Basic Statistics.

NGS Basic Statistics	
Total sequences	55640
Sequences flagged as poor quality	0
Sequence length	25-153
% GC	51
Filtered sequences (80 -85b)	21878
Percentage of filtered sequences (80 -85b)	39.32%
Abundance of sequences containing the SNP in the Top 10	14690
Percentage of sequences containing the SNP in the Top 10	99.5%

Table S-3. Next Generation Sequencing (NGS) results: Top 10 sequences.

Ranking	Abundance	Sequence
1	7635	5'-CAAAGAAATGGCCCTGATCCCCTGACTTCTT**T**AATGTGAGGG CATTTCTTGGTGCCCTATGGGCTCTCTGTGAAAAGCCAGG-3'
2	4348	5'-CAAAGAAATGGCCCTGATCCCCTGACTTCTT**T**AATGTGAGGG CATTTCTTGGTGCCCTGTGGGCTCTCTGTGAAAAGCCAGG-3'
3	2162	5'-CAAAGAAATGGCCCTGATCCCCTGACTTCTT**T**AATGTGAGGG CATTTCTTGGTGCTCTATGGGCTCTCTGTGAAAAGCCAGG-3'
4	125	5'-CAAAGAAATGGCCCTGATCCCCTGACTTCTT**T**AATGTGAGGG CATTTCTTGGTGCCTATGGGCTCTCTGTGAAAAGCCAGG-3'
5	113	5'-CAAAGAAATGGCCCTGATCCCCTGACTTCTT**T**AATGTGAGG CATTTCTTGGTGCCCTATGGGCTCTCTGTGAAAAGCCAGG-3'
6	83	5'-CAAAGAAATGGCCCTGATCCCTGACTTCTT**T**AATGTGAGGG CATTTCTTGGTGCCCTATGGGCTCTCTGTGAAAAGCCAGG-3'
7	77	5'-CAAAGAAATGGCCCTGATCCCCTGACTTCTT**T**AATGTGAGGG CATTTCTTGGTGCCTGTGGGCTCTCTGTGAAAAGCCAGG-3'
8	76	5'-CAAAGAAATGGCCCTGATCCCCTGACTTCTT**T**AATGTGAGGG CATTTCTTGGTGCCCTATGGGCTCTCTGTGAAAGCCAGG-3'
9	71	5'-CAAAGAAATGGCCCTGATCCCCTGACTTCTT**T**AATGTGAGGG CATTTCTTGGTGCCTCTGATGGCTCTCTGTGAAAAGCCAGG-3'
10	69	5'-CAAAGAAATGGCCCTGATCCCCTGACTTCTT**G**AATGTGAGGG CATTTCTTGGTGCCCTATGGGCTCTCTGTGAAAAGCCAGG-3'

*SNP is highlighted in red.

Table S-4. Comparison of our approach with other SNP detection methods reported in literature.

Amplification technique	Target	DNA extraction	Temperature	Detection Technique	Assay time	LOD	Applied to Real Samples	Ref.
Real-time PCR *(Cepheid Gene Xpert System's MTB/RIF assay)*	*Mycobacterium tuberculosis*	Chemical treatment	Cycling temperature	Fluorescence	Less than 2 h (detection)	4.5 genomes per reaction	Yes	(1)
Real-time quantitative PCR	BRAF V600E/K mutation	AllPrep DNA/RNA Mini kit and QIAamp DNA FFPE Tissue Kit (Qiagen, Hilden, Germany)	Cycling temperature	Fluorescence	Less than 2 h (detection)	Not specified	Yes	(2)
A combination of allele-specific PCR amplification and solid-phase hybridisation.	SNP related to attention-deficit hyperactivity disorder	PureLink Genomic DNA Mini Kit (Invitrogen) (Chemical lysis, DNA precipitation and centrifugation)	Cycling temperature	Optical, using Digoxigenin Antibody-HRP	210 min (DNA extraction: 50 min, amplification: 60 min, hybridisation: 60 min, detection: 40 min).	Not specified, (The sensor was used for detecting 12.5 ng of genomic DNA.)	Yes	(3)
Solid-Phase PCR on microspheres for minisequencing	SNP Minisequencing	Not specified	Cycling temperature	Gel electrophoresis	Days: (human genomic DNA hibridisation to the beads: 12–24 h, DNA amplification: 4-5 h, digestion of amplified DNA: two	Not specified, (The sensor was used for detecting 100–125 ng/µL	Yes	(4)

					overnight digestion procedures, sequencing procedure and purification: 1.5 h and overnight digestion of the beads before detection.)	denatured human genomic DNA.)		
Allele-specific Recombinase Polymerase Amplification	SNPs related to the treatment of tobacco addiction. SNPs: rs4680 (COMT gene), rs1799971 (OPRM1 gene), rs1800497 (ANKK1 gene), and rs16969968 (CHRNA5 gene)	PureLink Genomic DNA Mini Kit (Invitrogen) (Chemical lysis, DNA precipitation and centrifugation)	Isothermal (37°C)	Optical, using Digoxigenin Antibody-HRP	210 min (DNA extraction: 50 min, amplification: 60 min, hybridisation-detection: 100 min).	Not specified, (The sensor was used for detecting 2.6 ng of genomic DNA.)	Yes	(5)
Self-Powered Biosensor combined with DNA amplification	The 18-nucleotide (nt) sequence in the p53 gene comprising the mutation hotspot R273H	-	-	Electrochemistry	6.5 h aprox.	2 x 10^{-14} M<br>(Synthetic DNA)	No (Synthetic target spiked in cell lysates)	(6)
A combination of solid-phase primer extension mediated via RPA with biotinylated dNTPs	SNP located in the 14q12 locus of the Human β-myosin heavy chain (MYH7)	Thermal lysis,<br>Heating at 95 ^{o}C for 30 sec	Isothermal (37°C)	Colorimetric detection, using SA-Poly HRP	40.5 min (DNA extraction: 30 sec, amplification: 15 min, linking of SA-Poly HRP: 20 min, detection: 5 min).	3.6 x 10^{-13} M<br>(Synthetic DNA)	Yes	Our work

The prices of the reagents used in commercial DNA extraction methods are listed in Table S-5 and S-6 to demonstrate that just the cost of these reagents is almost comparable to our complete assay (where just the heating of sample is applied for DNA extraction).

Table S-5. Detailed cost analysis of the present approach

Solid-phase amplification	Price (€) /reaction 50 uL
Thiolated primers	0.12
Forward Primer	0.02
Biotin dCTP	0.48
Natural dNTPs	0.16
SA-poly-HRP	4.6-08
Maleimide plates	0.28
Skim milk	1.4E-08
RPA	2
**TOTAL**	3.06

Table S-6. Prices of commercial kits for DNA extraction from blood

Commercial kits for DNA extraction from blood	Price (€) /reaction
DNA Isolation Kit for Mammalian Blood	10.5
Invitrogen™ PureLink™ Genomic DNA Mini Kit	2.3
Cytiva illustra™ blood genomicPrep Mini Spin Kit	1.6
Invitrogen™ DNAzol™ BD Reagent, for isolation of genomic DNA from whole blood	2.5
Invitrogen™ Dynabeads™ DNA DIRECT™ Blood Kit	2.8
Invitrogen™ GeneCatcher™ gDNA Blood Kit, 3-10 mL	19.4
Thermo Scientific™ GeneJET Genomic DNA Purification Kit	2.3
Applied Biosystems™ MagMAX™ DNA Multi-Sample Ultra Kit	2.6
Thermo Scientific™ MagJET™ Whole Blood gDNA Kit	2.4

Supplementary Text

1. Double stranded DNA (dsDNA) generation from synthetic ssDNA to mimic genomic DNA

For dsDNA target preparation, a 100 µL PCR master mix was prepared containing 1x Dream Taq buffer, 200 nM of primers, 0.2 µM dNTPs, 1 U Dream Taq polymerase and 100 pM of synthetic ssDNA (Table S-1). The program used was based on an initial heating step of 95ºC for 2 min, followed by 25 rounds of PCR with 30 sec denaturation at 95ºC, 30 sec annealing at 60ºC and 30 sec elongation at 72ºC, with a final extension step at 72ºC for 5 min. The amplicon was purified using a DNA & Concentrator kit (Ecogen, Spain) and the final concentration was determined using the SimpliNano (ThermoFisher, Spain).

2. Assay time of the RPA reactions performed in liquid and solid phases

2.1 RPA reaction assay time in liquid phase:

RPA was performed according to the manufacturer's instructions (TwistAmp Liquid Basic kit, TwistDx, Cambridge, UK). Briefly, dsDNA was added to 50 µL of RPA reagents (1x Rehydration buffer, 1x Basic E-mix, 1x Core Reaction mix, 0.5 µM of primers (each of the four different reverse primers for individual reactions and the common forward primer), 0.2 mM dNTPs, 10 mM $Mg(OAc)_2$) for final 1 nM target concentration and incubated for 5, 15, 30 and 60 min at 37ºC. The samples were heated at 80ºC for 10 min to stop the RPA reaction.

Five microlitres of RPA amplified products were mixed with 4 µL of 6x Loading buffer and run in 2.6% (w/v) AGE prepared in 1x TBE buffer (Tris-Borate-EDTA, pH 8) at 110 mV for 20 min. The gel was pre-stained with GelRed nucleic acid stain (VWR, Spain) and imaged with a UV lamp (λ = 254 nm). The results are shown in Figure SI-1a.

2.2 RPA reaction assay time in solid phase:

Solid-phase primer extension assay was performed from primers immobilised on the surface of the wells of a maleimide activated microtiter plate. Using the same procedure described in the Experimental Section, the assay was performed at 5, 15, 30 and 60 min. The procedure was carried out both including (Figure SI-1b) or in the absence (Figure 2b, of the manuscript) of a denaturation step at basic pH, for comparison. Finally, this step, that consisted of adding 200 µL of 100 mM NaOH to each well for 2 minutes to remove/denature the remaining proteins from the RPA reaction, was applied to the final assay.

3. Study of the blood matrix effect on the recombinase polymerase-mediated solid phase primer extension

To study the matrix effect of the blood on recombinase polymerase-mediated solid phase primer extension, a random sequence which is not present in human genome *(M. tuberculosis mut rpoB, which carries the SNP C:*

*(5'*cgatcaaggagttcttcggcaccagccagctgagccaattcatggaccagaacaacccgctgtcggggttgacccacaagcgccgactgtcgg cgc***C***ggggcccggcggtctgtcacgtgagcgtgcc-*3') was selected.*

Firstly, the dsDNA was generated by PCR and purified following the same procedure described in Section 1 of Supporting Information, using the corresponding fully complementary reverse primer (5'-cacgtgacagaccgccgggcccc***G***-3') and the forward primer (*5'*-cgatcaaggagttcttcggc-3')). The obtained dsDNA was spiked on a fingerprick blood sample or in PBS (used as control) and the thermal treatment was applied.

Following the same procedure described in Experimental Section, the corresponding four reverse primers (5'-HS-ttt-cacgtgacagaccgccgggcccc***X***-3'; X = A, T, C and G, Positive primer G) were immobilised on the individual wells of the microtiter plate and using the forward Primer (*5'*-cgatcaaggagttcttcggc-3') the recombinase polymerase-mediated solid phase primer extension and colorimetric detection were carried out, as described in the Experimental Section of the manuscript. The results are shown in Figure S-4.

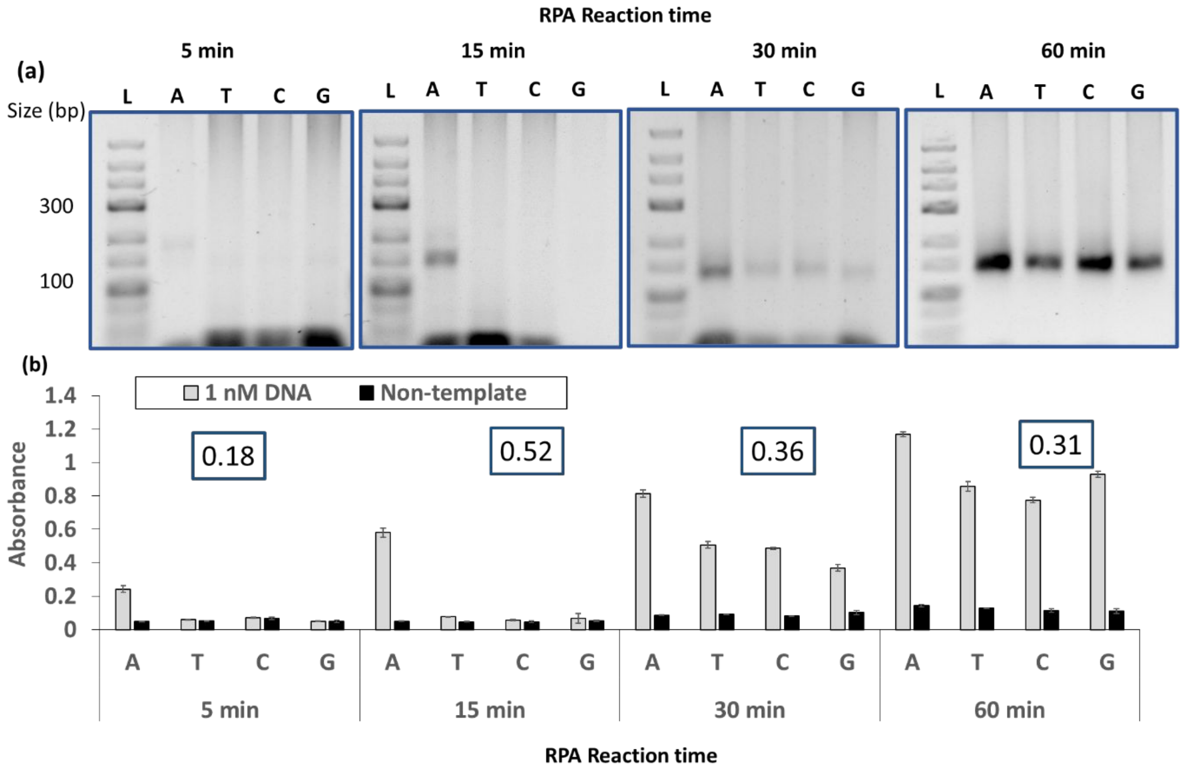

Figure S-1: Assay time of the RPA reaction performed in (a) liquid and (b) solid phase. (a) The 2.6% w/v agarose electrophoresis gels after the RPA reaction at different times show the increment of the intensity of the bands from non-specific primers with the reaction time. (b) The absorbance values recorded after recombinase polymerase-mediated solid phase primer extension also increase with the reaction time. The non-template controls were reduced with respect to those reported in Figure 2b by introducing a treatment at basic pH. (The difference between the signal from the specific primer and the media of the non-specific primer (Absorbance $(PA)_{Specific\ primer}$ - (Absorbance $(PT+PC+PG)_{Nonspecific\ primer}$ /3]) is reported in the insets for each time.

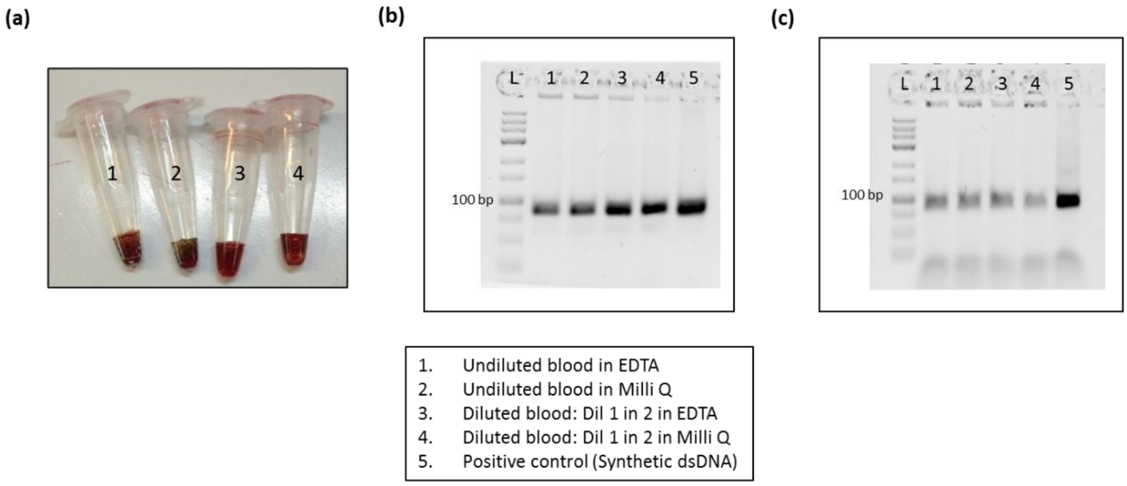

Figure S-2: Samples pre-treated using chemical lysis buffer to extract the human genomic DNA and further amplified: (a) A photograph showing the coagulation effect; electrophoresis gels after (b) PCR; (c) RPA.

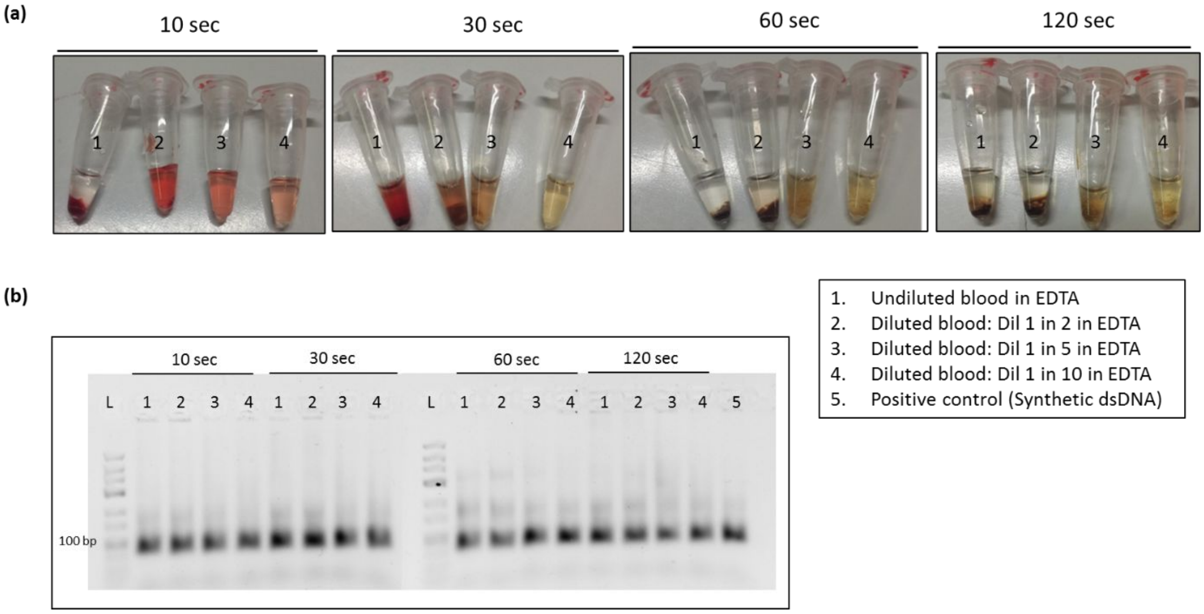

Figure S-3: Optimisation of heating time vs. dilution factor in samples pre-treated using thermal lysis to extract the human genomic DNA and further amplified: (a) Photographs showing the coagulation effect; (b) electrophoresis gels after RPA.

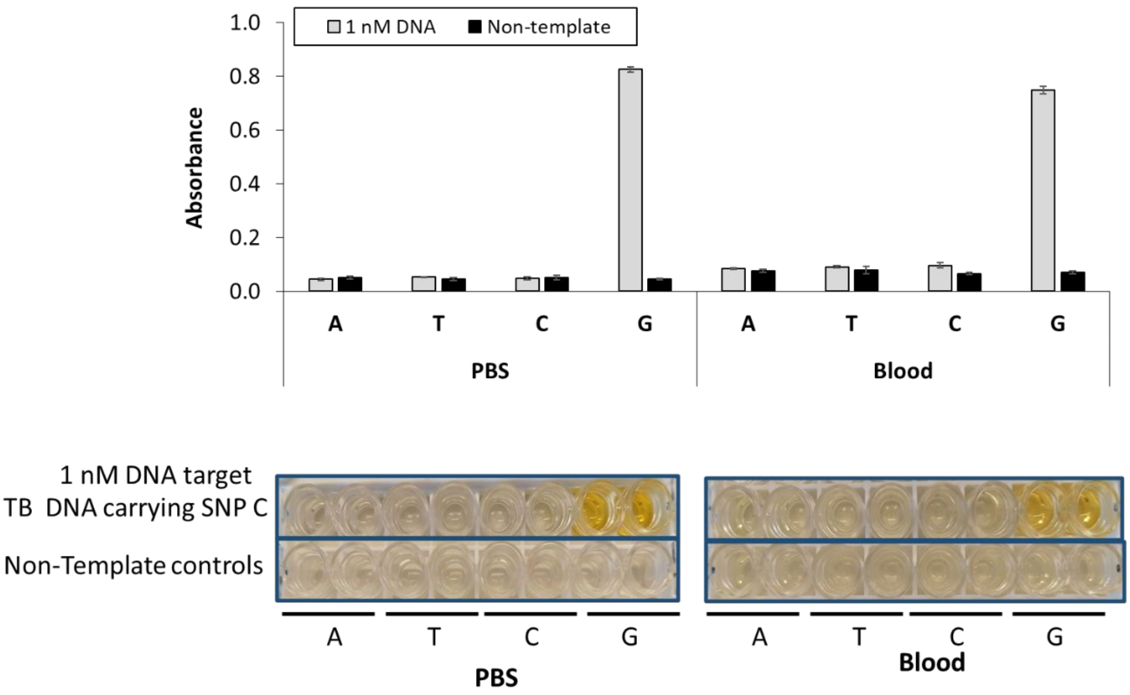

Figure S-4: Study of the blood matrix effect on the RPA reaction. RPA reaction was performed using a target sequence that is not present in the human genome (from *Mycobacterium tuberculosis*) spiked in PBS and in a blood sample. Positive primer G (SNP C).

References:

(1) Helb, D.; Jones, M.; Story, E.; Boehme, C.; Wallace, E.; Ho, K.; Kop, J.; Owens, M. R.; Rodgers, R.; Banada, P.; Safi, H.; Blakemore, R.; Lan, N. T. N.; Jones-López, E. C.; Levi, M.; Burday, M.; Ayakaka, I.; Mugerwa, R. D.; McMillan, B.; Winn-Deen, E.; Christel, L.; Dailey, P.; Perkins, M. D.; Persing, D. H.; Alland, D. Rapid Detection of Mycobacterium Tuberculosis and Rifampin Resistance by Use of On-Demand, near-Patient Technology. *J. Clin. Microbiol.* **2010**, *48* (1), 229–237. https://doi.org/10.1128/JCM.01463-09.

(2) Lung, J.; Hung, M.-S.; Lin, Y.-C.; Jiang, Y. Y.; Fang, Y.-H.; Lu, M.-S.; Hsieh, C.-C.; Wang, C.-S.; Kuan, F.-C.; Lu, C.-H.; Chen, P.-T.; Lin, C.-M.; Chou, Y.-L.; Lin, C.-K.; Yang, T.-M.; Chen, F. F.; Lin, P. Y.; Hsieh, M.-J.; Tsai, Y. H. A Highly Sensitive and Specific Real-Time Quantitative PCR for BRAF V600E/K Mutation Screening. *Sci. Rep.* **2020**, *10* (1), 16943. https://doi.org/10.1038/s41598-020-72809-7.

(3) Lázaro, A.; Yamanaka, E.; Maquieira, A.; Tortajada-Genaro, L. Allele-Specific Ligation and Recombinase Polymerase Amplification for the Detection of Single Nucleotide Polymorphisms. *Sensors Actuators B Chem.* **2019**, *298*, 126877. https://doi.org/10.1016/j.snb.2019.126877.

(4) Shapero, M. H.; Leuther, K. K.; Nguyen, A.; Scott, M.; Jones, K. W. SNP Genotyping by Multiplexed Solid-Phase Amplification and Fluorescent Minisequencing. *Genome Res.* **2001**, *11* (11), 1926–1934. https://doi.org/10.1101/gr.205001.

(5) Gu, C.; Kong, X.; Liu, X.; Gai, P.; Li, F. Enzymatic Biofuel-Cell-Based Self-Powered Biosensor Integrated with DNA Amplification Strategy for Ultrasensitive Detection of Single-Nucleotide

Polymorphism. *Anal. Chem.* **2019**, *91* (13), 8697–8704. https://doi.org/10.1021/acs.analchem.9b02510.

(6) Yamanaka, E. S.; Tortajada-Genaro, L. A.; Maquieira, Á. Low-Cost Genotyping Method Based on Allele-Specific Recombinase Polymerase Amplification and Colorimetric Microarray Detection. *Microchim. Acta* **2017**, *184* (5), 1453–1462. https://doi.org/10.1007/s00604-017-2144-0.
